# Proximate, intermediate, and distal predictors of under-five mortality in Chad: analysis of the 2014–15 Chad demographic and health survey data

**DOI:** 10.1186/s12889-020-09869-x

**Published:** 2020-12-07

**Authors:** Bright Opoku Ahinkorah, Abdul-Aziz Seidu, Eugene Budu, Ebenezer Kwesi Armah-Ansah, Ebenezer Agbaglo, Collins Adu, John Elvis Hagan, Sanni Yaya

**Affiliations:** 1 School of Public Health, Faculty of Health, University of Technology Sydney, Sydney, Australia; 2 Department of Population and Health, University of Cape Coast, Cape Coast, Ghana; 3 College of Public Health, Medical and Veterinary Sciences, James Cook University, Townsville, Queensland Australia; 4 Department of English, University of Cape Coast, Cape Coast, Ghana; 5 Department of Health Promotion and Disability Studies, Kwame Nkrumah University of Science and Technology, Kumasi, Ghana; 6 Department of Health, Physical Education, and Recreation, University of Cape Coast, Cape Coast, Ghana; 7 Neurocognition and Action-Biomechanics-Research Group, Faculty of Psychology and Sport Sciences, Bielefeld University, Bielefeld, Germany; 8 School of International Development and Global Studies, University of Ottawa, Ottawa, Canada; 9 The George Institute for Global Health, The University of Oxford, Oxford, UK

**Keywords:** Chad, Children, DHS, Global health, Public health, Under-five mortality

## Abstract

**Background:**

Under-five mortality in Chad reached a minimum value of 119 deaths per 1000 live births in 2018, compared with a maximum of 250 in 1972. Despite this decline in the  mortality trend, for every six children in Chad, one dies before the age of five. This study, therefore, investigated the proximate, intermediate, and distal determinants of under-five mortality in Chad.

**Methods:**

We used data from the 2014–15 Chad's Demographic and Health Survey. Data of 7782 children below 5 years were used for the study. Both descriptive and multivariable hierarchical logistic regression analyses were performed. Statistical significance was declared at *p* < 0.05.

**Results:**

Under-five mortality was found to be 130 deaths per 1000 live births in Chad, with variations across the various population sub-groups. For distal predictors, the likelihood of death was higher in children born in the FChari Baguirmi region (AOR = 3.83, 95% CI: 1.81–8.14). Children whose mothers belonged to the Baguirmi/Barma ethnic group (AOR = 8.04, 95% CI: 1.75–36.99) were more likely to die before the age of five. On the contrary, the likelihood of under-five mortality was low among children born in rural areas (AOR = 0.73, 95% CI: 0.55–0.97). With the intermediate predictors, the likelihood of under-five deaths was higher among children whose mothers had no formal education (AOR = 1.72, 95% CI: 1.06–2.77). Regarding the proximate predictors, the odds of under-five deaths was higher among male children (AOR = 1.03, 95% CI: 1.05–1.63) and first rank children (AOR = 1.58, 95% CI: 1.13–2.21).

**Conclusion:**

The study found that the determinants of under-five mortality in Chad are region of residence, place of residence, ethnicity, education, sex of child, and birth rank. These findings show that both socio-economic and proximate factors explain the disparities in under-five mortality in Chad. The identification of these factors can be pivotal towards the design of evidence-based interventions intended to improve child survival. Therefore, improving maternal education while refocusing and re-packaging existing strategies to target selected sub-regional populations with high under-five mortality is urgently required.

## Background

A collective programme of public health and international development agencies in recent times aims to reduce under-five mortality, defined as the death of children before their fifth birthday, because it is a key indicator of child and global health [1–3]. The Sustainable Development Goal three (SDG3) target two, for instance, aims at reducing the rates of under-five mortality to 25 or fewer deaths per 1000 live births by the year 2030 [4–6]. Under-five mortality is largely dependent on the quality of life and the wellbeing of the population, with many low- and middle-income countries, especially those in sub-Saharan Africa (SSA), experiencing a high number of these deaths [5, 7, 8]. Though the global under-five mortality rate has declined over 50% from 93 deaths to 39 deaths per 1000 live births between 1990 and 2018, the World Health Organisation (WHO) African region still remains vulnerable, with 76 deaths per 1000 live births, compared with 9 deaths per 1000 live births of WHO European region [7, 8].

Available evidence in SSA shows that 1 out of every 13 children dies before the age of 5 years due to malaria, measles, diarrhoea, respiratory infections, and other infections that are otherwise preventable through immunization [9]. The Republic of Chad ranks among the countries with the highest under-five mortality rates in SSA. With reference to Chad, under-five mortality reached its minimum value of 119 deaths per 1000 live births in 2018, compared with its maximum of 250 in 1972 [10]. Despite this decline in under-five mortality,  for every six children in Chad, one dies before the age of five because of diseases and malnutrition due to limited accessibility to child healthcare services such as immunisation, exclusive breastfeeding, and skilled birth attendance, antenatal care, and postnatal care [11]. Hence, in order to reduce under-five mortality in the country, it is essential to implement programmes focusing on expanded immunization, and early treatment and control of childhood diseases such as malaria and diarrhoea [7, 8, 12, 13].

In Chad, healthcare is provided through direct payment, free access to selected services, health insurance, and health mutual [11]. The out-of-pocket payment represents about half of the total health expenditure, with free access to some selected healthcare services entirely financed by the state. Other measures of gratuity are applied to chronic conditions such as malaria, AIDS, and tuberculosis. Health insurance is used by less than 2% of the people and usually contracted by large corporations for the benefit of their employees while health mutual is currently in its experimental phase in the southern regions. Health mutual is provided by private healthcare providers and non-governmental organisations. This scheme is in place to support the healthcare services provided by the government [11]. In spite of the efforts deployed by the state to improve health status in Chad, access to basic care remains a major challenge to most people due to socio-economic and geographical reasons [11]. For instance, as a consequence of the slow economic growth and high rural-urban disparities, many low-income African countries like Chad are living in abject poverty and in congested houses that do not have basic facilities like garbage disposal equipment, safe drinking water, and other sanitation facilities [14, 15]. This situation has led to the death of many people, including children under five years. Effective programs aimed at reducing under-five mortality in the country will require a better understanding of the factors associated with reported cases. Factors associated with under-five mortality in Chad have featured in some previous multi-country studies (e.g., Anyamele [16]; Yaya et al. [17]) although a special attention was not paid to Chad. One of these studies examined the predictors of under-five mortality using the theoretical framework developed for developing countries to analyze determinants of under-five mortality [17]. Although the variables considered in that study were proximate, intermediate, and distal factors, it is difficult to generalise the results to specific countries within sub-Saharan Africa which have different contexts, healthcare systems, and where children may have different risk exposures to under-five mortality. Using the theoretical framework developed for developing countries to analyze determinants of under-five mortality as used in the study by Yaya et al. [17], the present study aims to determine the proximate, intermediate, and distal determinants of under-five mortality in Chad. Examining how proximate, intermediate, and distal factors are associated with under-five mortality in Chad will provide a comprehensive literature for public health and policy interventions aimed at reducing under-five mortality in the country.

## Methods

### Data

Data for this study came from the 2014–15 Chad Demographic and Health Survey (DHS), which is part of the MEASURE DHS Program, aimed at obtaining information on a number of population, health, and nutrition-related issues, such as under-five mortality. For this study, we used the birth recode file, which had information on all births. Data of 7782 children below 5 years constituted the sample size. The inclusion criteria was women who had given birth within a period of 5 years prior to the survey and had complete information on all the variables considered in this study. Taking households as sampling units, the survey employed a multi-stage stratified sampling design to select all eligible women for the interviews. Two strata were created in each field (urban and rural). Taken together, 626 primary sampling units (PSUs) or clusters were systematically selected from the list of enumeration areas that were predefined during the 2009 General Population and Housing Census. Households in each cluster constituted the list from which eligible households were selected, with 25 households per cluster in the urban locations and 30 households per cluster in rural locations. Again, 17, 965 households from 4075 urban areas in 163 clusters and 13,890 rural households nested in 463 clusters were selected. All resident women aged 15–49 years or those present during the night preceding the survey were eligible to be interviewed [18]. The details of the methodology can be found in the 2014–15 Chad Demographic and Health Survey report [18].

### Study variables

Under-five mortality, defined as the death of children before their fifth birthday, was considered the outcome variable for the study. We then recoded it as either “0” (No) or “1” (Yes). For the explanatory variables, the conceptual framework for the study of child survival in developing countries was drawn from Mosley and Chen [19], which focused on three main categories of variables (proximate, intermediate, and distal factors). The proximate factors were sex of child, birth size, birth rank and birth interval, age of mother at child birth, antenatal care visit, place of delivery, and delivery assistance. The intermediate factors were wealth index, mother’s ethnicity, mother’s religion, mother and partner's level of education as well as mother and partner's occupation. Region and place of residence were considered as distal factors. Some of the variables were recoded as follows: educational level of the mother and the father (0 = No education, 1 = Primary education, 2 = Secondary and higher education); religion (1 = Christianity, 2 = Islam, 3 = Others); type of occupation of mother and father (0 = Not working, 1 = Official [professional, technical, managerial and clerical], 2 = Sales and services, 3 = Agricultural, 4 = Manual); age of mother at child birth (1 = < 20 years, 2 = above 20 years of age); antenatal service received by the mother (0 = No and 1 = Yes, any visit); place of delivery (0 = Home and 1 = Health facility), and birth attendance during delivery (0 = By Traditional Birth Attendant/other and 1 = By Skill Birth Attendant or health professional). Birth rank and birth interval were recoded as first birth rank, 2–3 birth rank & = < 2 years of birth interval, > = 4 birth rank & > 2 years of birth interval and > = 4 birth rank & = < 2 years of birth interval. However, the original coding in the dataset for variables such as sex of child, birth size, wealth index, mother’s ethnicity, region, and place of residence was maintained.

### Data analysis

Three steps were followed in analysing the data. For the first step, frequency tables were generated to describe the proportions of all the explanatory variables. This procedure was followed by a distribution of under-five mortality per the explanatory variables with their respective confidence intervals (CIs). A bivariate logistic regression analysis on both the explanatory and outcome (under-five mortality) variables was conducted with the aim of examining the association between all the potential determinants and under-five mortality, without adjusting for other covariates during the second step. The results were presented as unadjusted odds ratios (UOR) at 95% confidence interval. The third step involved a multivariable hierarchical logistic regression analysis to examine the distal, intermediate, and proximate determinants of under-five mortality. Preceding these procedures, the variance inflation factor (VIF) was employed to conduct a multicollinearity test on all the statistically significant variables. This test showed no evidence of collinearity among the explanatory variables (Mean VIF = 1.46, Max VIF = 2.10, Minimum = 1.00**)**. The multivariable hierarchical logistic regression analysis which was performed in three stages was carried out using all the explantory variables irrespective of their significant association with under-five mortality at the bivariate analysis level. At the first stage, the community level distal socio-economic variables were entered in the first model to assess their association with under-five mortality (see Model I). The household and individual level socio-economic variables were added to the first model at the second stage to assess their association with the community level distal socio-economic determinant variables and under-five mortality (see Model II). The third stage involved the addition of the proximate determinant variables to the community level distal socio-economic determinant variables and the household and individual level socio-economic variables. The results were presented using adjusted odds ratios (AORs) at 95% confidence interval. The pseudo R^2^ was used to assess goodness-of-fit of logistic models. The STATA version 14.2 was used for data cleaning, management, and analysis. An applied sample weight (v005/1000,000) was also performed to correct for over- and under-sampling. The SVY command was used to account for the complex survey design and generalizability of the findings. The weighted results from the analysis are reported in the study.

## Results

### Descriptive and bivariate logistic regression on the distribution of under-five mortality per socio-demographic characteristics

This study includes a total of 7782 children under five years. Table 1 shows results on the distribution of under-five mortality per the characteristics of the sample. Majority of the children were from the Hadjer-lamis region (8.0%), rural areas (81.5%), poorer wealth quintile households (22.0%), Peulh/Foulbe ethnic group (27.9%), and Muslim background (54.1%). Approximately, 51% of the children were males, 28.1% were larger than average size at birth, and 52.1% were born with a > = 4 birth rank, and > 2 years of birth interval.

**Table 1:** Under-five mortality rate (per 1000 live births), 5 year periods preceding the survey and unadjusted odds ratio by explanatory variables (*n* = 7782, weighted)

Determinants	Frequency (n)	Percentage (%)	Under-five mortality rate [95% CI]	UOR [95% CI]
**Region**				
Betha	299	3.8	121 [84–158]	2.62^*^ (1.14–6.01)
Borkou/Tibesti	30	0.4	99 [59–139]	3.85^**^ (1.68–8.79)
FChari baguirmi	395	5.1	171 [140–203]	3.69^***^ (1.74–7.80)
Guera	468	6.0	113 [79–147]	1.53 (0.65–3.62)
Hadjer-lamis	622	8.0	103 [88–178]	2.91^**^ (1.35–6.23)
Kanem	320	4.1	86 [56–116]	1.75 (0.76–4.00)
Lac	417	5.4	101 [80–122]	1.93 (0.85–4.39)
Logone occidental	462	5.9	179 [159–198]	2.38^*^ (1.06–5.37)
Lagone oriental	704	9.1	187 [151–223]	3.67^***^ (1.70–7.91)
Mandoul	539	6.9	131 [109–153]	2.47^*^ (1.11–5.49)
Mayo kebbi est	612	7.9	143 [115–171]	1.92 (0.85–4.32)
Mayo kebbi oust	445	5.7	123 [96–151]	0.91 (0.36–2.25)
Moyen chari	388	5.0	139 [111–168]	2.30^*^ (1.00–5.28)
Ouaddai	435	5.6	101 [73–130]	1.89 (0.81–4.42)
Salamat	163	2.1	133 [104–161]	1.00 (0.38–2.62)
Tandjile	541	6.9	171 [144–200]	2.90^**^ (1.34–6.30)
Wadi fira	218	2.8	53 [40–66]	1
N’djamena	441	5.7	133 [107–160]	2.43^*^ (1.10–5.34)
Barh el gazel	106	1.4	67 [48–86]	1.23 (0.49–3.06)
Ennedi	35	0.5	91 [61–120]	1.68 (0.69–4.10)
Sila	142	1.8	84 [60–109]	2.06 (0.87–4.89)
**Place of residence**				
Urban	1439	18.5	132 [112–152]	1
Rural	6343	81.5	133 [124–142]	0.78 (0.61–1.00)
**Wealth index**				
Poorest	1584	20.4	137 [116–155]	1.35 (0.97–1.88)
Poorer	1713	22.0	144 [128–160]	1.18 (0.85–1.64)
Middle	1629	20.9	122 [107–137]	0.92 (0.65–1.30)
Richer	1597	20.5	121 [108–135]	1
Richest	1260	16.2	123 [110–149]	1.50^*^ (1.08–2.09)
**Ethnicity**				
Gorane	109	1.4	90 [69–110]	3.89^*^ (1.20–12.66)
Arab	463	6.0	104 [82–125]	3.29^*^ (1.00–10.83)
Baguirmi/Barma	833	10.7	232 [151–313]	11.28^***^ (3.02–42.07)
Kanembou/Bornou	80	1.0	112 [92–132]	3.82^*^ (1.17–12.48)
Boulala/Medego	781	10.0	133 [97–169]	4.67^*^ (1.32–16.61)
Ouadai/Maba	275	3.5	106 [82–131]	2.86 (0.85–9.67)
Zaghawa/Bideyat	631	8.1	56 [27–85]	1
Dadajo/Kibet	75	1.0	134 [103–166]	4.66^*^ (1.36–16.00)
Bidio/Migami	212	2.7	138 [107–170]	4.29^*^ (1.16–18.80)
Mounding	218	2.8	77 [56–97]	1.14 (0.23–5.73)
Massa/Mousseye	203	2.6	140 [101–178]	4.03^*^ (1.15–14.19)
Toupouri/Kera	403	5.2	112 [76–148]	1.24 (0.20–7.50)
Sara	179	2.3	162 [143–181]	5.42^**^ (1.70–17.26)
Peul/Foulbe	2169	27.9	143 [113–175]	6.20^***^ (1.76–21.88)
Tema/Assongori	195	2.5	63 [34–91]	2.74 (0.68–11.12)
Gabri/Kabalaye	123	1.6	184 [127–242]	7.40^**^ (1.92–28.52)
Marba/Lele	362	4.6	148 [98–198]	4.03^*^ (1.12–14.47)
Mesmedje/massalat	66	0.9	94 [24–164]	5.88^*^ (1.28–27.02)
Karo/Xime	116	1.5	186 [135–236]	2.61 (0.58–11.86)
Achit/banda/kim	289	3.7	141 [111–170]	3.53 (0.98–12.65)
**Religion**				
Christianity	3307	42.5	157 [141–172]	1.26^*^ (1.01–1.57)
Islam	4208	54.1	113 [102–123]	1
Others	268	3.4	124 [92–155]	0.64 (0.30–1.38)
** Educational level of mother**				
No education	5137	66.0	124 [114–134]	1.20 (0.80–1.80)
Primary	1777	22.8	159 [135–182]	1.44 (0.92–2.25)
Secondary /Higher	868	11.2	111 [86–135]	1
**Educational level of partner**				
No education	4494	57.7	124 [112–137]	0.85 (0.65–1.11)
Primary	1514	13.5	156 [140–172]	0.98 (0.69–1.38)
Secondary /Higher	1774	22.8	126 [108–144]	1
**Mother’s occupation**				
Not working	3733	48.0	117 [104–129]	1.50 (0.21–11.05)
Official	50	0.6	107 [8–205]	1
Sales and services	2624	33.7	154 [141–167]	2.04 (0.28–15.01)
Agricultural	1271	16.3	126 [107–145]	1.44 (0.19–10.75)
Manual	104	1.3	108 [50–166]	2.67 (0.32–22.01)
**Partner's occupation**				
Official	455	5.8	94 [68–121]	1
Sales and services	1645	21.1	117 [102–132]	1.11 (0.67–1.84)
Agricultural	4942	63.5	135 [125–147]	0.98 (0.61–1.59)
Manual	740	9.5	152 [129–176]	1.31 (0.75–2.30)
**Size of child at birth**				
Very large	1605	20.6	110 [87–133]	1
Larger than average	2182	28.1	128 [112–144]	1.04 (0.75–1.45)
Average	2045	26.3	135 [116–154]	1.04 (0.75–1.44)
Smaller than average	959	12.3	134 [115–165]	1.04 (0.70–1.53)
Very small	990	12.7	160 [120–200]	1.06 (0.73–1.53)
**Sex of the child**				
Male	3974	51.1	140 [130–150]	1.32^*^ (1.07–1.64)
Female	3808	48.9	120 [112–128]	1
**Birth rank and birth interval**				
First birth rank	1180	15.1	134 [117–151]	1.64^**^ (1.18–2.28)
2–3 birth rank & = < 2 years of birth interval	693	8.9	141 [123–160]	1.18 (0.77–1.79)
> = 4 birth rank & > 2 years of birth interval	4050	52.1	102 [91–113]	1.11 (0.84–1.46)
> = 4 birth rank & = < 2 years of birth interval	1859	23.9	110 [95–125]	1
**Age of mother at childbirth**				
< 20 years	5784	74.3	133 [125–141]	1.17 (0.92–1.50)
More than 20 years	1998	25.7	122 [108–137]	1
**Antenatal care visit**				
No	2875	36.9	120 [85–154]	1.05 (0.85–1.30)
Yes	4907	63.1	91 [74–107]	1
**Place of delivery**				
Home	6056	77.8	132 [119–144]	1
Health facility	1726	22.2	127 [104–149]	1.38^*^ (1.08–1.76)
**Delivery assistance**				
By TBA/Others	4887	62.8	129 [120–138]	0.71^**^ (0.57–0.87)
By SBA/Health professional	2895	37.2	131 [114–149]	1
National total	7782	–	130 [122–138]	

Source: Authors’ computation from the 2014–15 Chad DHS data

*COR* crude Odds Ratio, *CI* Confidence Interval, *TBA* Traditional birth attendants, *SBA* Skilled birth attendants

* *p*<0.05, ** *p*<0.01, *** *p*<0.001

Under-five mortality was found to be 130 deaths per 1000 live births in Chad, with variations across the various population sub-groups. Under-five mortality was 179 per 1000 live births in the Logone occidental region, compared to 53 deaths per 1000 live births in the Wadi Fira region. There was no much urban (132 deaths per 1000 live births) and rural (133 deaths per 1000 live births) variations in under-five mortality in the country. Children from poorer households experienced a higher under-five mortality rate (144 deaths per 1000 live births), compared to those from richer households (121 deaths per 1000 live births). Under-five mortality rate was higher among children who were very small at birth (160 deaths per 1000 live births), compared to those who were large at birth (110 deaths per 1000 live births). Male children had higher under-five mortality rate (140 deaths per 1000 live births) than females (120 deaths per 1000 live births). Under-five mortality was higher among children with 2–3 birth rank and = < 2 years of birth interval (141 deaths per 1000 live births), compared to those with > = 4 birth rank and > 2 years of birth interval (102 deaths per 1000 live births).

The unadjusted logistic regression results show that in terms of the distal factors, the likelihood of death was higher in children born in the FChari baguirmi region (UOR = 3.69, 95% CI: 1.74–7.80), compared to those born in the Wadi fira region. With the intermediate factors, children born to women of richest wealth quintile were more likely to die compared to those born to children of richer wealth quintile (UOR = 1.50, 95% CI: 1.08–2.09). Children whose mothers belonged to the Baguirmi/Barma ethnic group were more likely to die before the age of five, compared to those whose mothers belonged to the Zaghawa/Bideyat ethnic group (UOR = 11.28, 95% CI: 3.02–42.07). Children born to Christian mothers also had higher odds of dying compared to those born to Muslim women (UOR = 1.26, 95% CI: 1.01–1.57). In terms of the proximate determinants, the risk of death was high among male children (UOR = 1.32, 95% CI: 1.07–1.64), first birth rank children (UOR = 1.64, 95% CI: 1.18–2.28), children born in health facilities (UOR = 1.38, 95% CI: 1.08–1.76) compared to female children, children with > = 4 birth rank & = < 2 years of birth interval, and children born at home. Conversely, there was lower odds of death among children delivered by TBA/others compared to those delivered by SBA/Health professionals (UOR = 0.71, 95% CI: 0.57–0.87).

### Multivariable hierarchical logistic regression analysis on distal determinants of under-five mortality

Table 2 shows results of the multivariable hierarchical logistic regression analysis. Model I assessed the relationship between all the distal variables and under-five mortality. For the distal predictors, the likelihood of death was higher in children born in the FChari baguirmi region (AOR = 3.83, 95% CI: 1.81–8.14), compared to those born in the Wadi fira region. Children whose mothers belonged to the Baguirmi/Barma ethnic group (AOR = 8.04, 95% CI: 1.75–36.99) were more likely to die before the age of five, compared to those whose mothers belonged to the Zaghawa/Bideyat ethnic group. Contrarily, the likelihood of under-five mortality was low among children born in rural areas, compared to those born in urban areas (AOR = 0.73, 95% CI: 0.55–0.97). With the intermediate predictors, the likelihood of under-five deaths was high among children whose mothers had no formal education (AOR = 1.72, 95% CI: 1.06–2.77), compared to those whose mothers had secondary/higher education. Regarding the proximate predictors, the odds of under-five deaths was high among male children (AOR = 1.03, 95% CI: 1.05–1.63) and first rank children (AOR = 1.58, 95% CI: 1.13–2.21), compared to female children and children with > = 4 birth rank & = < 2 years of birth interval.

**Table 2:** Multivariable logistic regression results for determinants of under-five mortality in the 5 years preceding the survey-adjusted odds ratio

Determinants	Model I			Model II			Model III		
	AOR	95% CI		AOR	95% CI		AOR	95% CI	
**Region**									
Betha	2.64^*^	1.15	6.06	1.89	0.60	6.01	1.84	0.58	5.82
Borkou/Tibesti	3.50^**^	1.54	7.97	2.61	0.75	9.01	2.46	0.71	8.53
FChari baguirmi	3.83^**^	1.81	8.14	2.19	0.72	6.67	2.04	0.67	6.25
Guera	1.49	0.63	3.53	0.57	0.16	1.94	0.57	0.17	1.94
Hadjer-lamis	2.93^*^	1.36	6.29	2.23	0.74	6.72	2.24	0.74	6.78
Kanem	1.77	0.77	4.05	1.35	0.39	4.63	1.32	0.39	4.76
Lac	1.96	0.86	4.46	1.39	0.39	4.94	0.83	0.24	2.84
Logone occidental	2.36^*^	1.05	5.31	0.86	0.25	2.91	1.35	0.38	4.76
Lagone oriental	3.70^***^	1.71	7.98	1.39	0.42	4.53	0.84	0.24	2.83
Mandoul	2.50^*^	1.12	5.56	0.90	0.27	3.03	1.45	0.39	5.48
Mayo kebbi est	1.93	0.86	4.35	1.56	0.42	5.77	1.45	0.39	5.48
Mayo kebbi oust	0.92	0.37	2.28	0.54	0.14	2.07	0.51	0.13	1.99
Moyen chari	2.28	0.99	5.23	0.89	0.26	3.07	0.88	0.25	3.03
Ouaddai	1.86	0.80	4.35	1.47	0.53	4.10	1.40	0.50	3.96
Salamat	0.96	0.37	2.53	0.55	0.16	1.87	0.54	0.16	1.84
Tandjile	2.94^**^	1.36	6.40	1.26	0.35	4.50	1.21	0.34	4.36
Wadi fira	1			1			1		
N’djamena	1.87	0.83	4.22	1.05	0.34	3.19	0.96	0.31	2.93
Barh el gazel	1.26	0.50	3.13	0.96	0.26	3.50	0.94	0.26	3.42
Ennedi	1.64	0.67	4.00	1.65	0.50	5.41	1.53	0.47	4.96
Sila	2.04	0.86	4.84	1.02	0.33	3.16	1.01	0.32	3.15
**Place of residence**									
Urban	1			1			1		
Rural	0.73^*^	0.55	0.97	0.85	0.58	1.25	0.88	0.59	1.31
**Wealth index**									
Poorest				1.25	0.88	1.79	1.25	0.87	1.80
Poorer				1.14	0.80	1.61	1.11	0.78	1.60
Middle				0.92	0.64	1.31	0.93	0.65	1.32
Richer				1			1		
Richest				1.50	0.97	2.33	1.47	0.94	2.29
**Ethnicity**									
Gorane				3.29	0.89	12.12	3.36	0.91	12.35
Arab				2.82	0.71	11.16	2.67	0.68	10.50
Baguirmi/Barma				8.04^**^	1.75	36.99	8.95_**_	1.96	40.90
Kanembou/Bornou				3.54	0.89	14.03	3.55	0.90	14.01
Boulala/Medego				3.46	0.80	14.95	3.49	0.82	14.48
Ouadai/Maba				2.69	0.69	10.44	2.73	0.71	10.57
Zaghawa/Bideyat				1			1		
Dadajo/Kibet				6.81^**^	1.62	28.70	6.96^**^	1.67	29.01
Bidio/Migami				7.28^*^	1.58	33.65	7.42^**^	1.63	33.81
Mounding				2.29	0.33	16.08	2.56	0.37	18.00
Massa/Mousseye				2.78	0.60	12.83	3.07	0.66	14.18
Toupouri/Kera				0.87	0.11	6.79	1.00	0.13	7.88
Sara				4.99^*^	1.07	23.26	5.23^*^	1.13	24.16
Peul/Foulbe				5.43^*^	1.26	23.45	5.44^*^	1.27	23.25
Tema/Assongori				3.25	0.69	15.31	3.22	0.69	14.99
Gabri/Kabalaye				5.57	0.99	31.39	6.15^*^	1.10	34.33
Marba/Lele				3.43	0.65	18.24	3.69	0.69	19.61
Mesmedje/massalat				5.13	0.94	28.07	5.35	0.97	29.47
Karo/xime				4.02	0.63	25.53	4.68	0.73	30.18
Achit/banda/kim				3.26	0.75	14.10	3.36	0.79	14.40
**Religion**									
Christianity				1.37	0.72	2.61	1.20	0.73	2.71
Islam				1			1		
Others				1.30	0.49	3.44	1.30	0.47	3.58
**Mother’s educational level**									
No education				1.72^*^	1.06	2.77	1.89^*^	1.16	3.10
Primary				1.55	0.97	2.48	1.70^*^	1.06	2.74
Secondary/Higher				1			1		
** Partner's educational level**									
No education				0.94	0.66	1.34	0.98	0.68	1.40
Primary				0.89	0.61	1.29	0.93	0.63	1.35
Secondary/Higher				1			1		
**Mother's Occupation**									
Not working				1.28	0.17	9.45	1.22	0.16	9.24
Official				1			1		
Sales and services				1.59	0.21	11.87	1.57	0.21	12.00
Agricultural				1.46	0.20	11.00	1.44	0.19	11.08
Manual				2.57	0.30	21.86	2.29	0.26	19.91
** Partner's occupation**									
Official				1			1		
Sales and services				1.15	0.67	1.96	1.09	0.63	1.86
Agricultural				1.02	0.60	1.73	0.97	0.57	1.67
Manual				1.21	0.67	2.17	1.11	0.62	1.99
**Sex of the child**									
Male							1.03^*^	1.05	1.63
Female							1		
**Size of the child at birth**									
Very large							1		
Larger than average							1.11	0.79	1.56
Average							1.24	0.88	1.75
Smaller than average							1.22	0.81	1.85
Very small							1.22	0.81	1.85
**Birth rank and birth interval**									
First birth rank							1.58^**^	1.13	2.21
2–3 birth rank & = < 2 years of birth interval							1.16	0.76	1.77
> = 4 birth rank & > 2 years of birth interval							1.06	0.80	1.41
> = 4 birth rank & = < 2 years of birth interval							1		
**Age of mother at childbirth**									
< 20 years							1.13	0.87	1.45
More than 20 years							1		
**Antenatal care visit**									
No							1.22	0.93	1.60
Yes							1		
**Place of delivery**									
Home							1		
Health facility							1.15	0.79	1.67
**Delivery assistance**									
By TBA/Others							0.76	0.55	1.05
By SBA/Health professional							1		
Pseudo R^2^	0.020			0.036			0.045		

Source: Authors’ computation from the 2014–15 Chad DHS data

*AOR* adjusted Odds Ratio, *CI* Confidence Interval, *TBA* Traditional birth attendants, *SBA* Skilled birth attendants

Model I adjusted for distal factors

Model II adjusted for distal and intermediate factors

Model III adjusted for distal, intermediate and proximate factors

## Discussion

Chad, like many low- and middle-income countries, is currently experiencing fast socio-demographic, economic, and population health shifts. However, there seems to be variations at the sub-national level on the cited indices in the country that might affect their public health policy. Using data from the nationally representative survey, 2014–15 Chad DHS, this study found that the odds of children dying before their fifth birthday was high in Chad (130 deaths per 1000 live births). Region of residence, place of residence, ethnicity, education, sex of child, and birth rank and interval were identified as predictors of under-five mortality in Chad.

The majority of under-five deaths occurred among children in the FChari Baguirmi, Lagone Oriental region, Borkou/Tibesti, Tandjile, and Hadjer-lamis, compared to those in Wadi fira. This finding confirms previous studies on the association between regional variations and under-five mortality in other low- and middle-income countries (e.g., Ghana and India [20, 21]). The regional differentials noted might be connected with spatial inequality and variations in social development in these regions, with possibly varying population density, regional development, political as well as economic resources [22]. Surprisingly, children in rural areas had lower odds of under-five mortality, compared to those in urban areas although the statistical significance of this association was attenuated after controlling for confounders in successive models. This finding contradicts the commonly established assumption from numerous researches conducted in Kenya [23], Nigeria [24, 25], Ghana [26], and sub-Saharan Africa as a whole [27] that infants born in urban areas have better access to healthcare services and other necessary health-related services that are critical for child survival and development. Chad is a country with a greater percentage (77%) of its population in rural areas [28]. From this premise, it is possible that most rural areas in Chad are endowed with natural environmental conditions like fresh agriculture products and fresh water sources that are healthier. Comparatively, urban dwellers may be confronted with highly polluted water sources, unhygienic processed food products, overcrowding, and intermixing household occupancies as well as poor sanitary conditions. Therefore, mothers and their young children in urban areas may be highly exposed to these unhealthier conditions that are associated with morbidity and mortality, compared to their rural counterparts.

The study further established that under-five mortality varied by the sex of a child, with male children having higher odds of dying before their fifth birthday, compared with female children. Several studies globally support this finding [17, 29–32]. Principally, some biological and social reasons have been cited to account for this finding. Biologically, male children are highly susceptible to infections (e.g., neonatal disorders) and are more likely to be born premature. Besides, they have a larger average body size and big head circumference which prolong the time of their mothers’ delivery period [17, 31, 33]. Socially, in some societies, gender discrimination (e.g., feeding and medical care practices) exists among male and female children, with the practices favouring females [17, 31, 33]. The study also established statistically significant association between birth rank and under-five mortality, a finding established in previous studies [24, 34–36].

Other findings suggest that children born to mothers with no and low formal education (primary) are more likely to die before their fifth birthday, compared to those whose mothers have secondary/higher education. Plethora of research evidence indicate that mother’s education has a significant association with child survival, which works via both direct and indirect pathways [24, 32, 37–40]. Mothers who are more educated on good childcare practices are more likely to have prompt healthcare-seeking behaviours for their children [37–40]. These knowledgeable mothers are also more likely to offer appropriate and timely feeding to their children and observe other hygienic behaviours associated with good health outcomes. Educated mothers, unlike uneducated ones, are also more empowered to take certain decisions against bad socio-cultural practices [41]. Unlike uneducated mothers, educated mothers are also more likely to be empowered economically to afford certain basic necessities (such as child food supplements) and other indirect cost associated with childcare [41, 42]. Similar to previous studies in Nigeria [43, 44], sub-Saharan Africa [45], and 36 low- and middle-income countries [46], ethnic variations exist in the association of child mortality in Chad. Specifically, children from Baguirmi/Barma ethnic group had higher odds of under-five mortality, compared to children from Zaghawa/Bideyat ethnic group. The probable explanations for this finding could be the differences in ethno-cultural practices or compositions (e.g., early age of marriage, pubertyrites), socio-demographic characteristics, and socio-economic conditions among the ethnic groups [45, 47].

### Strengths and limitations of the study

The strength of this study lies in the use of nationally representative data which support the generalizability of the findings in Chad. The large sample size, high response rate, and the use of valid survey and rigorous statistical methods provide trustworthiness to the obtained results. Similarly, the use of a wide range of multiple hierarchical variables warrant deeper examination of these numerous factors.

Despite these strengths, there are some limitations inherent in this study. First, the cross-sectional nature of the data does not allow us to draw causal inferences but only associations. Second, the outcome variable, under-five mortality, was collected based on retrospective self-report from the mothers, which can distort the accuracy of the results. This study design subjects the data to social desirability and recall bias. For example, some mothers, in their report, may classify stillbirths as under-five mortality. There is also the possibility of underreporting since mothers may find it difficult to reveal information on those unfortunate events in their lives. We also acknowledge that the division of risk factors of child mortality into proximate, intermediate, and distal factors may be arbitrary. For instance, sometimes a region, generally understood as a distal factor, may be a proximate risk factor. The causal chain analysis of health outcomes also includes physiological and pathophysiological causes. However, in this study, the risk factors for under-five mortality did not included these causes since the Chad DHS dataset did not have variables on these factors.

## Conclusion

The study found that the determinants of under-five mortality in Chad are region of residence, place of residence, ethnicity, education, sex of child, and birth rank. These findings show that both socio-economic and proximate factors explain the disparities in under-five mortality in Chad. The findings provide a basis for considering proximate, intermediate, and distal determinants concurrently when dealing with under-five mortality. The identification of these factors can be pivotal towards the design of evidence-based interventions (e.g., adult education programs, socio-economic empowerment through entrepreneurial training, health education and promotion programs) intended to improve child survival. Strategically, improving maternal education while re-focusing and re-packaging existing strategies to target selected sub-regional populations with under-five mortality is urgently required. Our findings on the wide disparities in the child mortality rates in different regions of Chad is essential for public health and policy interventions. This is because, since Chad is a country with limited financial resources, regional disparities in under-five mortality will help government and non-governmental organisations to channel resources to regions which need most attention when it comes to addressing under-five mortality. Future research should investigate the improvements in child mortality over time, especially against ethno-cultural practices in Chad. Undertaking such studies could help inform appropriate strategies that are most pertinent to the epidemiology of child survival in the country.

## Data Availability

The dataset can be accessed at https:// https://dhsprogram.com/data/dataset

## Abbreviations

AOR: Adjusted Odds Ratio
CI: Confidence Interval
DHS: Demographic and Health Survey
VIF: Variance Inflation Factor
PNC: Post-natal check-up
SDG: Sustainable Development Goal
LMICs: Low- and Middle-Income Countries
SSA: sub-Saharan Africa
